# Predicting sentinel lymph node metastasis in melanoma patients: A machine learning-based predictive model

**DOI:** 10.1016/j.jdin.2025.09.012

**Published:** 2025-10-09

**Authors:** Hengxiang Zhang, Hanbin Wang, Shida Zhang, Tianwen Gao, Yu Liu, Chunying Li, Weinan Guo

**Affiliations:** a Department of Dermatology, Xijing Hospital, Fourth Military Medical University, Xi'an, Shaanxi, China; b Innovation Research Institute, Xijing Hospital, Fourth Military Medical University, Xi'an, Shaanxi, China; c Military Medical Innovation Center, Fourth Military Medical University, Xi'an, Shaanxi, China

**Keywords:** artificial intelligence, machine learning, melanoma, neural network, predictive model, sentinel lymph node biopsy

## Abstract

**Background:**

Risk factors for sentinel lymph node (SLN) metastasis in melanoma have been studied. However, there remains a lack of widely applicable models with considerable predictive potential for clinical use.

**Objective:**

To developed a well-performing machine learning-based model for predicting SLN metastasis in melanoma patients.

**Methods:**

This study collected data on 351 melanoma patients with sentinel lymph node biopsy from our center. Univariate and multivariate logistic regression was used for recognizing key features. The optimal model was selected from 10 machine learning algorithms based on the F1 score. SHapley Additive exPlanations was employed to interpret the outcome of the predictive model. R package Shiny was used to develop a web tool.

**Results:**

The neural network model was chosen with the highest F1-score (0.73), indicating considerable predictive accuracy and calibration. SHapley Additive exPlanations results indicate the related factors for SLN metastasis in melanoma patients were Breslow thickness, microsatellites, Ki67 index, and subtype. Ultimately, we developed a web-based tool to promote the clinical application of the model.

**Limitations:**

Retrospective study, single institution.

**Conclusions:**

This study established a robust and interpretable machine learning approach for melanoma SLN metastasis prediction. With high sensitivity and accuracy, this approach could reduce misdiagnosis rates and alleviate patient suffering.

**Capsule Summary**

• Nonsubungual melanoma, greater Breslow thickness, higher Ki67 index, and the present of microsatellites are risk factors for positive sentinel lymph node metastasis.
• By inputting variables, the web-based neural network model can compute and interpret the predicted probability for sentinel lymph node metastasis, with high accuracy, sensitivity and conveniency.

## Introduction

Sentinel lymph node biopsy (SLNB) serves as a cornerstone in the staging and prognostic evaluation of melanoma patients. By identifying early lymphatic metastasis, SLNB not only refines tumor-node-metastasis staging,^1^ but also informs personalized treatment strategies and predicts long-term survival outcomes.^2^ Current National Comprehensive Cancer Network clinical guidelines recommend SLNB for patients with intermediate-thickness melanomas (Breslow Thickness (BT) ≥1.0 mm) or low-thickness melanomas (BT ≥0.8 mm) but high-risk histopathological features.^3^ However, existing studies highlight that up to 80% of SLNB in patients who have intermediate-thickness melanoma yield negative results,^4^^,^^5^ exposing patients to unnecessary surgical risks and health care costs. Furthermore, a critical limitation of current decision-making frameworks lies in their reliance on the experience of pathologists, which lack standardized quantitative thresholds and are heavily dependent on subjective interpretation.^6^

Clinically, features such as BT, age, mitotic rate, ulceration, and tumor-infiltrating lymphocyte density have shown correlations with the result of SLNB in preliminary studies.^5^, ^6^, ^7^ These indicators are well-suited for outcome prediction; however, these studies failed to provide quantifiable predictive tools. Current literature provides no validated machine learning (ML)-based predictive model and web-based calculator tailored for predicting sentinel lymph node (SLN) metastasis in melanoma populations.^8^ This gap limits the leap of from the discovery of clinical phenomena to guiding clinical practice.

Advances in ML and deep learning provide unprecedented opportunities to address this challenge. Unlike traditional statistical models, ML algorithms excel at capturing nonlinear relationships between high-dimensional variables and outcomes, enabling robust predictions even with heterogeneous datasets.^9^ With the support of ML algorithms, we developed a well-performing model using a relatively small dataset, which confirms that our selected feature variables are indeed closely associated with outcomes. Additionally, by integrating SHapley Additive exPlanations (SHAP) values,^10^ we enhance the model’s transparency, enabling clinicians to visualize how individual features contribute to predictions. The finalized model will be deployed as an open-access web tool. By harmonizing clinical expertise with artificial intelligence, this work advances precision oncology and sets a precedent for deploying interpretable ML tools in routine practice.

## Method

### Study design and data collection

**Fig 1:**
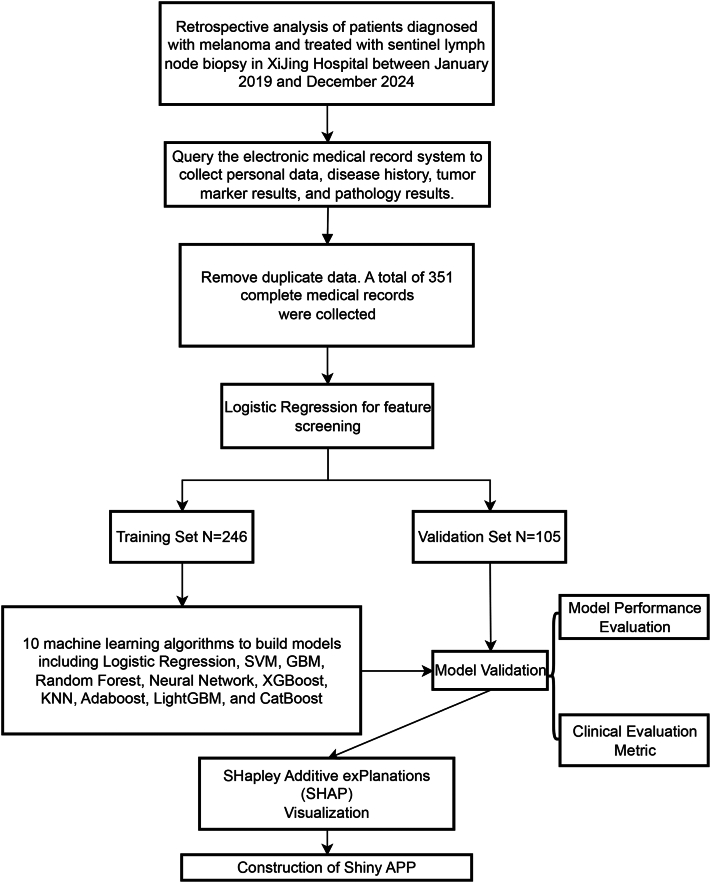
Flow chart of the study. This schematic outlines the study design and analytical pipeline for the development of machine learning-based prognostic models in melanoma patients undergoing sentinel lymph node biopsy. The study retrospectively included patients treated at Xijing Hospital between January 2019 and December 2024. Personal, clinical, and pathological data were extracted from electronic medical records, resulting in 351 nonduplicated complete record. Feature selection was performed using logistic regression. The cohort was subsequently partitioned into training (*n* = 246) and validation (*n* = 105) sets. Ten distinct machine learning algorithms—including Logistic Regression, Support Vector Machine (SVM), Gradient Boosting Machine (GBM), Random Forest, neural network, Exterme Gradient Boosting (XGBoost), k-Nearest Neighbors (KNN), AdaBoost, Light Gradient Boosting Machine (LightGBM), and Categorical Boosting (CatBoost)—were employed for model construction. Models were rigorously evaluated using standard performance metrics, validated internally, and interpreted via SHapley Additive exPlanations (SHAP) for feature importance visualization. A Shiny web application was subsequently developed to facilitate clinical translation and real-time prediction.

This study adhered to the guidelines established for the Transparent Reporting of a Multivariable Prediction Model for Individual Prognosis or Diagnosis.^11^ The overall workflow chart was illustrated in Fig 1.

Many models in this field employ the principle of 10 events per variable to evaluate the effective sample size.^12^ This principle requires a minimum ratio of 10 observed events per predictor variable.^13^ Details of variable encoding are presented in the Supplementary Table I, available via Mendeley at https://data.mendeley.com/datasets/gw4c35mzpn/1. Pathological data were collected by professional pathologists who were blinded to the purpose of the collection. The first value was used for analysis if any data was repeated. The clinical data were reviewed and collected by 2 experienced physicians using a standardized data collection form independently.

### Statistical analyses

Statistical analyses were conducted using the R programming language (version 4.2, http://www.R-project.org). A *P* value of less than .05 was considered significant (2-tailed). The “mice” package in R was used to impute the missing data.^14^ The R caret package was utilized to randomly divide the 351 patients into a training set comprising 246 participants and a validation set consisting of 105 participants for internal validation, adhering to the theoretical ratio of 7:3 (Supplementary Table III, available via Mendeley at https://data.mendeley.com/datasets/gw4c35mzpn/1). More detailed method of Winsorization and imputation will be provided in Supplementary Materials, available via Mendeley at https://data.mendeley.com/datasets/gw4c35mzpn/1.

### ML-based model construction and assessment

This study aimed to investigate the performance of 10 ML algorithms—Random Forest, Exterme Gradient Boosting, Categorical Boosting, Light Gradient Boosting Machine, Support Vector Machine, Gradient Boosting Machine, AdaBoost, neural network (NN), and K-nearest neighbors. Ten cross-validation procedures were conducted to identify the optimal configurations for 10 ML models. More detailed information about model construction and validation will be provided in Supplementary Materials, available via Mendeley at https://data.mendeley.com/datasets/gw4c35mzpn/1.

### SHAP analysis and Shiny APP construction

While ML models have demonstrated strong predictive accuracy in numerous studies, their opaque decision-making processes often hinder interpretability. SHAP offers a robust framework for elucidating the “black box” behavior of ML algorithms.^15^ The web-based application was implemented using R Shiny package and incorporates the optimal predictive model.

## Results

In this cohort of 351 patients with melanoma (Table I), significant differences were observed between those with SLNB^-^ cases (*n* = 218) and SLNB^+^ cases (*n* = 133). Patients in SLNB^+^ group had higher BT (mean [SD], 3.7 [1.9] mm vs 3.0 [2.1] mm; *P* < .001), higher Ki67 proliferation index (34.3 [17.7]% vs 26.9 [16.2]%; *P* < .001), and higher rates of microsatellites (29.5% vs 16.3%; *P* = .007). Tumor subtype distribution also differed significantly (11.3% vs 24.8% for the predominant nonsubungual type; *P* = .003), while the level of mitotic rate showed marginal variation (*P* = .008). Neither *BRAF*, *NRAS*, nor *c-KIT* gene mutations were found to be associated with SLN metastasis, but this result may be limited by the sample size. Moreover, no significant correlation was observed between lymphovascular invasion, ulceration, tumor-infiltrating lymphocytes, or Clark level and SLN metastasis in our cohort (detailed variable encoding specifications are provided in Supplementary Table I, available via Mendeley at https://data.mendeley.com/datasets/gw4c35mzpn/1).

**Table I:** Baseline characteristics of all patients

Parameters	Levels	SLNB^-^ (*N* = 218)	SLNB^+^ (*N* = 133)	*P* value
Gender	0	115 (53%)	68 (51.1%)	.819∗
	1	102 (47%)	65 (48.9%)	
Age	Mean ± SD	58.2 ± 12.6	56.5 ± 13.4	.232†
Subtype	0	164 (75.2%)	118 (88.7%)	.003∗
	1	54 (24.8%)	15 (11.3%)	
Site	1	191 (87.6%)	105 (78.9%)	.093∗
	2	21 (9.6%)	21 (15.8%)	
	3	6 (2.8%)	7 (5.3%)	
Breslow_thickness	Mean ± SD	3.0 ± 2.1	3.7 ± 1.9	<.001†
Ulcer	0	64 (29.8%)	30 (23.8%)	.288∗
	1	151 (70.2%)	96 (76.2%)	
Ki67	Mean ± SD	26.9 ± 16.2	34.3 ± 17.7	<.001†
Microsatellites	0	174 (83.7%)	86 (70.5%)	.007∗
	1	34 (16.3%)	36 (29.5%)	
Lymphovascular_invasion	0	187 (89%)	114 (91.2%)	.657∗
	1	23 (11%)	11 (8.8%)	
TIL	0	185 (90.2%)	106 (86.9%)	.45∗
	1	20 (9.8%)	16 (13.1%)	
Clark_level	1	5 (2.6%)	0 (0%)	.14∗
	2	4 (2%)	1 (0.8%)	
	3	26 (13.3%)	10 (8.5%)	
	4	140 (71.4%)	88 (74.6%)	
	5	21 (10.7%)	19 (16.1%)	
Mitotic_rate	0	8 (4.2%)	3 (2.6%)	.008∗
	1	30 (15.7%)	8 (6.9%)	
	2	36 (18.8%)	15 (12.9%)	
	3	62 (32.5%)	33 (28.4%)	
	4	44 (23%)	43 (37.1%)	
	5	11 (5.8%)	14 (12.1%)	
*BRAF* mutation	0	47 (66.2%)	29 (53.7%)	
	1	24 (33,8%)	25 (46.3%)	.233∗
*NRAS* mutation	0	47 (79.7%)	29 (60.4%)	
	1	12 (20.3%)	19 (39.6%)	.957∗
*c-KIT* mutation	0	47 (88.7%)	20 (58.8%)	
	1	6 (11.3%)	14 (41.2%)	.484∗
Type	1	190 (88.8%)	105 (82%)	.392∗
	2	11 (5,1%)	10 (7.8%)	
	3	3 (1.4%)	2 (1.6%)	
	4	8 (3.7%)	7 (5.5%)	
	5	2 (0.9%)	4 (3.1%)	

*SD*, Standard division; *SLNB*, sentinel lymph node biopsy; *TIL*, tumor infiltrating lymphocyte.

∗ Chi-square test/Fisher’s exact test.

† Independent sample *t*-test.

We assume that there is multicollinearity among the variables selected through baseline analysis, and we further explore its causal relationship with the outcomes. Univariate analysis (Table II) identified BT (odds ratio [OR]uni = 1.25 [1.11-1.40], *P* < .001), Ki67 (ORuni = 1.03[1.01-1.04], *P* < .001), microsatellites (ORuni = 2.02[1.19-3.40], *P* = .009), subtype (ORuni = 0.51[0.32-0.81], *P* = .004). Using multivariate logistic regression analysis with a threshold of *P* < .05, we finally identified 4 significant characteristic variables, including BT (ORmulti = 1.14 [1.01-1.29], *P* = .034), Ki67 (ORmulti = 1.02 [1.01-1.04], *P* = .001), microsatellites = 1 (ORmulti = 1.86 [1.07-3.23], *P* = .004) and subtype = 1 (ORmulti = 0.50 [0.31-0.81], *P* = .005).

**Table II:** Univariate and multivariate logistic regression analysis for the relationship between risk factors and SLNB result

Parameter	Desc	SLNB^−^ (*N* = 218)	SLNB^+^ (*N* = 133)	OR (univariable)	OR (multivariable)
Gender	0	116 (53.2%)	68 (51.1%)		
	1	102 (46.8%)	65 (48.9%)	1.09 (0.71-1.67, *P* = .705)	
Age	Mean ± SD	58.2 ± 12.6	56.5 ± 13.4	0.99 (0.97-1.01, *P* = .215)	
Breslow_thickness	Mean ± SD	3.0 ± 2.1	3.9 ± 1.8	1.25 (1.11-1.40, *P* < .001)	1.14 (1.01-1.29, *P* = .034)
Subtype	0	122 (56%)	95 (71.4%)		
	1	96 (44%)	38 (28.6%)	0.51 (0.32-0.81, *P* = .004)	0.50 (0.31-0.81, *P* = .005)
Site	1	191 (87.6%)	105 (78.9%)		
	2	21 (9.6%)	21 (15.8%)	1.82 (0.95-3.48, *P* = .071)	
	3	6 (2.8%)	7 (5.3%)	2.12 (0.70-6.48, *P* = .186)	
Ulceration	0	65 (29.8%)	34 (25.6%)		
	1	153 (70.2%)	99 (74.4%)	1.24 (0.76-2.01, *P* = .391)	
Ki67	Mean ± SD	26.8 ± 16.0	34.3 ± 17.6	1.03 (1.01-1.04, *P* < .001)	1.02 (1.01-1.04, *P* = .001)
Mitotic_rate	0	9 (4.1%)	4 (3%)		
	1	34 (15.6%)	9 (6.8%)	0.60 (0.15-2.39, *P* = .464)	
	2	39 (17.9%)	16 (12%)	0.92 (0.25-3.43, *P* = .905)	
	3	73 (33.5%)	36 (27.1%)	1.11 (0.32-3.85, *P* = .870)	
	4	52 (23.9%)	52 (39.1%)	2.25 (0.65-7.77, *P* = .200)	
	5	11 (5%)	16 (12%)	3.27 (0.80-13.35, *P* = .098)	
Microsatellites	0	183 (83.9%)	96 (72.2%)		
	1	35 (16.1%)	37 (27.8%)	2.02 (1.19-3.40, *P* = .009)	1.86 (1.07-3.23, *P* = .028)
Lymphovascular_invasion	0	193 (88.5%)	121 (91%)		
	1	25 (11.5%)	12 (9%)	0.77 (0.37-1.58, *P* = .470)	
TIL	0	198 (90.8%)	117 (88%)		
	1	20 (9.2%)	16 (12%)	1.35 (0.68-2.72, *P* = .394)	
Clark_level	3	28 (13.4%)	11 (8.3%)		
	4	154 (73.7%)	100 (75.8%)	1.65 (0.79-3.47, *P* = .184)	
	5	27 (12.9%)	21 (15.9%)	1.98 (0.80-4.87, *P* = .137)	

*OR*, Odds ratio; *SD*, standard division; *SLNB*, sentinel lymph node biopsy; *TIL*, tumor infiltrating lymphocyte.

To further eliminate the potential impact of multicollinearity on subsequent analyses, we calculated the variance inflation factor among the key features. The results indicated that the variance inflation factor for all features were less than 5 (Supplementary Fig 1, available via Mendeley at https://data.mendeley.com/datasets/gw4c35mzpn/1), suggesting that there is no multicollinearity among the selected features, allowing for further analysis.

The analysis included 351 eligible patients, who were randomly assigned into training (70%, *n* = 246) and validation (30%, *n* = 105), with the validation data being completely independent from the training set. Then we developed and validated 10 ML-based models for predicting SLN metastasis in melanoma patients. By conducting 10 cross-validations, the optimal configuration was identified for 10 ML models using the training dataset.

**Fig 2:**
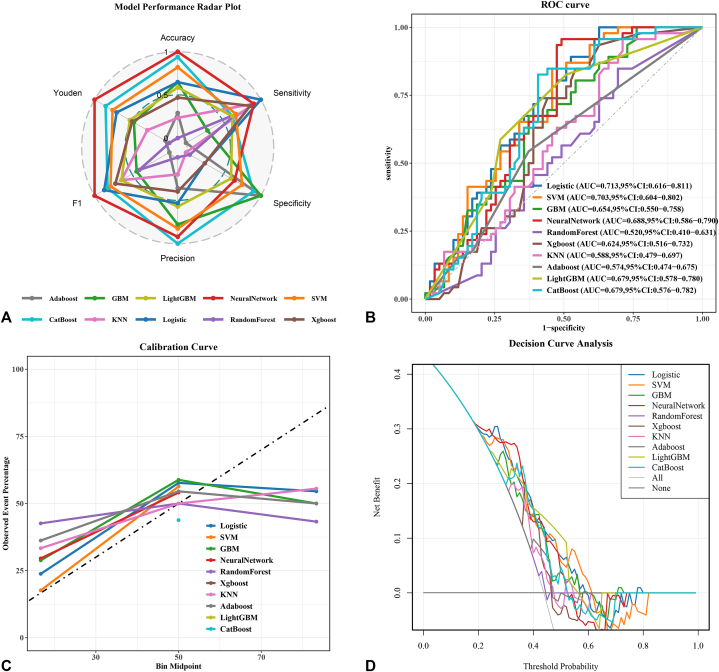
Model performance evaluation. **A,** Radar chart of evaluation metrics across 10 models. A multiaxis radar plot compares the performance of 10 machine learning models—including AdaBoost, Support Vector Machine (SVM), Gradient Boosting Machine (GBM), Random Forest, neural network, Exterme Gradient Boosting (XGBoost), k-Nearest Neighbors (KNN), Logistic Regression, Light Gradient Boosting Machine (LightGBM), and Categorical Boosting (CatBoost)—across 6 key metrics: accuracy, sensitivity, specificity, precision, F1 score, and Youden’s index. The plot highlights distinct performance profiles, with larger polygon areas indicating stronger overall discriminative ability. **B,** Receiver operating characteristic (ROC) curves. ROC curves depict the diagnostic performance of each model by plotting the true positive rate (sensitivity) against the false positive rate (1 − specificity). Models are ranked by area under the curve (AUC). **C,** Calibration curves for predicted probabilities. Calibration curves compare the models’ predicted probabilities of positive outcomes with observed event frequencies. The dashed diagonal represents ideal calibration. **D,** Decision curve analysis (DCA) evaluating clinical utility. DCA illustrates the net benefit of each model across a range of probability thresholds (0-1). The teal *horizontal line* assumes all patients are positive (“treat all”), while the *gray line* assumes none are positive (“treat none”).

The NN model demonstrated superior performance in validation set, achieving highest F1-score (0.73) (Fig 2, *A*), the relatively higher area under the receiver operating characteristic curve (0.689) (Fig 2, *B*), and lowest Brier score (0.226), indicating excellent predictive accuracy and calibration (Fig 2, *C*). Decision curve analysis curves also showed that patients predicted by the model could obtain significant clinical benefit (Fig 2, *D*). Through performance evaluation, our model demonstrated a good detection rate for low-risk positive patients for its highest Youden index (0.465), which is a necessary capability in predicting the risk of tumor metastasis.

**Fig 3:**
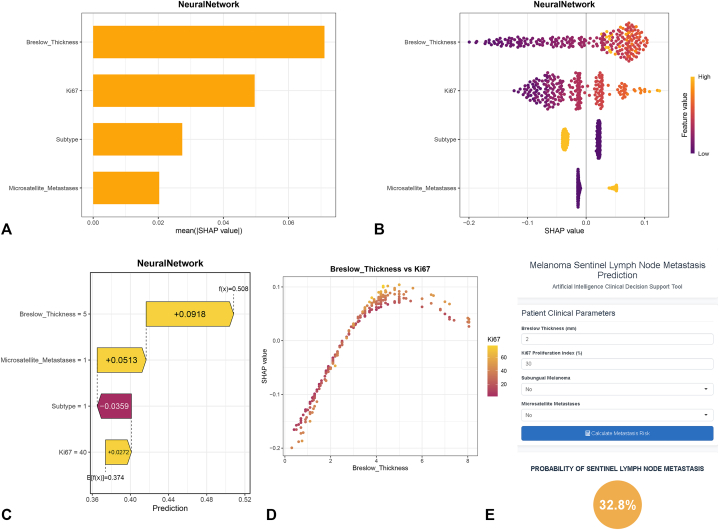
Model interpretability analysis and clinical deployment interface of the neural network-based prognostic prediction model. **A,** SHAP summary plot: feature importance ranking. Horizontal bar chart illustrating the mean absolute SHAP (SHapley Additive exPlanations) values for the top 4 features derived from the neural network model, ranked in descending order of global importance. “Breslow_Thickness” demonstrates the highest feature importance, followed by “Ki67”, “Subtype”, and “Microsatellite_Metastases”. The length of each bar corresponds to the magnitude of the feature's average impact on model output. **B,** SHAP beeswarm plot: distribution of feature effects. Beeswarm plot visualizing the distribution of SHAP values (x-axis) for each feature (y-axis). Each point represents an individual prediction instance. The color gradient from purple (low feature value) to yellow (high feature value) illustrates the direction and strength of the relationship between feature values and their contribution to the prediction outcome. Features are ordered by global importance as in **(A)**. **C,** SHAP waterfall plot: explanation of an individual prediction. Local interpretability plot deconstructing the model's prediction for a single instance. The baseline model output (expected value) is 0.374. Each *horizontal bar* indicates the contribution of a specific feature value, driving the prediction upward (red) or downward (blue) to arrive at the final predicted value of 0.508. This plot provides a transparent, additive explanation of how the model generated this specific prognosis. **D,** Bivariate correlation scatter plot: Breslow Thickness vs Ki67. Scatter plot exploring the relationship between 2 key predictive variables: “Breslow_Thickness” (x-axis) and “Ki67” (y-axis). Point color corresponds to the value of “Ki67” (purple: low, yellow: high), visually reinforcing a positive correlation between these histopathological markers. This relationship aligns with their high feature importance rankings in the predictive model. **E,** Web interface for the AI-based clinical decision support tool. Screenshot of the interactive web application developed for clinical deployment. The user interface allows input of key clinical parameters (eg, “Breslow Thickness”, “Ki67”). Clicking the “Calculate Metastasis Risk” button (blue) triggers the model, with the prediction result (32.8%), facilitating real-time, interpretable risk assessment at the point of care.

Previous SLN metastasis prediction models, such as the Melanoma Institute Australia (MIA)^16^ model and the Memorial Sloan Kettering Cancer Center (MSKCC)^17^ model, have constructed nomograms but did not provide further explanations for the models. To elucidate the prediction process of the models, we employed SHAP analysis. Feature importance rankings were visualized through SHAP analysis for the NN model (Fig 3, *A*), which was further complemented by SHAP bee swarm plot (Fig 3, *B*). As shown in the figure, the most important variable for outcome prediction is BT, followed by Ki67, subtype, and microsatellites. The bee swarm plot intuitively illustrates how the direction of changes in each variable affects the direction of the predictions: for instance, as BT increases, the corresponding SHAP value also increases (the probability of SLNB^+^ increases). We then used an example to show how individual predictions can be explained (Fig 3, *C*). Furthermore, the influence of factor-level features on the predictive model's risk between “Breslow Thickness” and “Ki67 index” was assessed using SHAP dependency plots, as illustrated in (Fig 3, *D*). The dependency plots among the remaining variables are presented in Supplementary Fig 3, *A-D*, available via Mendeley at https://data.mendeley.com/datasets/gw4c35mzpn/1.

We performed a comparative analysis of the MSKCC, MIA, and NN models on an independent internal validation set. The NN model (area under the curve [AUC] = 0.688, 95% CI: 0.586-0.790) demonstrated superior discriminative ability over both the MSKCC model (AUC = 0.681, 95% CI: 0.578-0.785) and the MIA model (AUC = 0.538, 95% CI: 0.422-0.654). A radar plot comparing model performance across 6 evaluation metrics revealed that the NN covered the largest area, indicating more robust overall performance. Furthermore, the NN model achieved the lowest Brier score (0.2263) and provided the highest net benefit across most threshold probabilities in decision curve analysis curves (Supplementary Fig 4, *A-D*, available via Mendeley at https://data.mendeley.com/datasets/gw4c35mzpn/1). Finally, we developed a web-based prediction tool using Shiny to facilitate practical risk assessment (Fig 3, *E*), which could be available at https://melanomaslnmodel.shinyapps.io/Shiny-/

## Discussion

This study focuses on SLN metastasis in melanoma, a topic of significant interest in the field. Previous research has been limited to conventional models, providing only nomograms for variable importance interpretation. Investigators at the MSKCC developed a nomogram using 5 prognostic factors, including age, BT, ulceration, Clark level of invasion, and tumor location, that achieved an AUC of 0.694 with a total of 979 patients who underwent successful SLN biopsy for cutaneous melanoma.^17^ More recently, Lo et al^16^ at MIA developed a nomogram using 6 prognostic factors (age, BT, melanoma subtype, ulceration, tumor mitotic rate, and lymph vascular invasion) from 3496 patients and reported an AUC of 0.739. Compared with linear models, ML provides objective classification criteria, thereby enhancing the reliability and efficacy of evaluations.^18^ Among which, NN offer distinct advantages in medical prediction tasks by automatically extracting complex patterns from raw data like genomic sequences and clinical notes.^19^ Their deep architectures excel at modeling intricate nonlinear relationships in high-dimensional medical data, outperforming traditional linear models and shallow algorithms. With transfer learning enabling effective small-sample learning and end-to-end optimization reducing error propagation, NN demonstrate particular strengths in developing medical predictive models.^20^

Clinically, features such as BT, age, mitotic rate, ulceration, and tumor-infiltrating lymphocyte density have shown correlations with the result of SLNB in preliminary studies.^5^, ^6^, ^7^ However, few studies have focused on acral melanoma (particularly subungual morphology) and selected Ki67 proliferation index as a variable feature. Subungual melanoma (SUM) is a type of malignant melanoma more prevalent in Asians and associated with poor prognosis.^21^ Interestingly, our findings suggest that SUM exhibit a paradoxically lower risk of SLN metastasis. We question whether this abnormal phenomenon is a distinctive characteristic of Asian individuals. However, results from a single-institution study at Oregon Health & Science University^22^ including SUM and nonsubungual acral melanoma patients who underwent SLNB consists with our study, with the SLNB positive rate lower in SUM compared with non-subungual acral melanoma patients (7.4% vs 22.9%, *P* = .07). Meanwhile, both Ki67 index and mitotic rate serve as established biomarkers for evaluating tumor cell proliferation.^23^ Ki67 spans the entire cell cycle (G1/S/G2/M phases), it provides a more accurate assessment of overall tumor proliferative activity.^24^ Moreover, multiple studies have demonstrated that high Ki67 expression is significantly associated with lymph node metastasis.^25^^,^^26^ In this study, correlation analysis revealed that mitotic rate was significantly associated with SLNB outcomes (*P* = .008). However, this variable was not selected in univariate or multivariate logistic regression analyses (*P* > .05). To ensure model performance and stability, we excluded this variable. During data reanalysis, we identified “Microsatellites/Microsatellitosis” as one of the characteristic variables for SLN metastasis. Defined as one or more discontinuous nests of melanoma cells measuring at least 0.3 mm in diameter and separated from the primary lesion by more than 0.05 mm of normal tissue, the presence of microsatellites constitutes a criterion for *N*-stage classification in melanoma according to AJCC guidelines.^27^ Existing studies have demonstrated microsatellites as independent risk factors for reduced overall survival, melanoma-specific survival, and disease-free survival, with hazard ratios of 1.57, 1.76, and 1.76, respectively.^28^ Additionally, melanomas with microsatellites exhibit higher frequencies of local clinical metastasis (14% vs 3%).^29^ One study revealed that SLN metastases are a significant prognostic factor in patients with microsatellites.^30^ However, no prior research has established whether melanomas with microsatellites are more prone to SLN metastasis. Our study addresses this knowledge gap by utilizing this variable to replace the original one in model construction, thereby enhancing both interpretability and reliability.

The presence of ulceration is frequently associated with poor prognosis in melanoma and has been demonstrated by multiple studies as a correlate of SLN metastasis.^7^^,^^31^ Unlike in Caucasian populations, acral melanoma predominates as the primary subtype in East Asian cohorts, exhibiting a notably worse prognosis.^32^^,^^33^ In our cohort, the ulceration incidence exceeded 70% regardless of SLNB results, substantially higher than reported in previous studies (MSKCC: 30.5%; MIA: 29.6%). Mitotic rates were also elevated, with 77.6% of patients exhibiting >4 mitoses/mm^2^ (MIA: 54.5%). Additionally, while both MSKCC and MIA studies identified age as a risk factor for SLN metastasis, this was not validated in our study. We attribute these discrepancies to potential population-specific variations.^34^

To further validate the clinical value of our model, we conducted comparative analyses of the MSKCC, MIA, and NN models on an independent internal validation set. The results demonstrated that the NN model outperformed both the MSKCC and MIA models across all evaluation metrics (Supplementary Fig 4, *A-D*, available via Mendeley at https://data.mendeley.com/datasets/gw4c35mzpn/1). Additionally, unlike previous studies relying solely on linear models, our approach involved constructing and comparing 10 distinct models to identify the optimal variable combination, with SHAP analysis elucidating feature contributions and inter-relationships. The final web-based deployment ensures practical clinical accessibility, enabling real-time risk stratification and facilitating integration into routine diagnostic workflows. Among the 10 models evaluated, the NN model exhibited the most balanced performance and was consequently selected for online deployment. Among models with F1 scores >0.7, the Logistic model achieved the highest sensitivity (1.000), while the Categorical Boosting model attained the highest specificity (0.593). The provision of tailored model selections for distinct clinical needs represents another key contribution of this study.

This study has several limitations that should be acknowledged. First, the retrospective design inherently limits the strength of evidence compared to prospective studies. Secondly, our model development and validation were conducted internally based on data availability. While this allowed for thorough model optimization in a relatively controlled environment, it inherently limits the model's portability to other institutions. Extensive external validation would provide a more rigorous assessment of model robustness, though noise or biases in external datasets may obscure the true performance identified through internal validation.^35^^,^^36^ In addition, the relatively small sample size in this study limited the number of variables we could incorporate into the predictive model, consequently affecting its performance. This necessitated the use of sophisticated algorithms to enhance performance within limited data—improvements that are ultimately constrained. Future research should focus on large-scale, multicenter validation to strengthen the robustness of our findings.^37^

## Conclusion

We recommend clinicians consider key parameters including BT, Ki67 index, subtype, and microsatellites when determining the necessity of SLNB. Our web-based tool is available to provide efficient diagnostic guidance during this decision-making process.

Looking ahead, we aim to enhance our risk assessment model by integrating comprehensive clinical and pathological data and hope more research teams will focus on this field and seek opportunities for multicenter clinical studies for further validation. This approach will ultimately improve prognostic accuracy while maintaining clinical practicality for widespread adoption.

### Declaration of generative AI and AI-assisted technologies in the writing process

During the preparation of this work the authors used DeepSeek R1 in order to improve language and readability. After using this tool/service, the authors reviewed and edited the content as needed and take full responsibility for the content of the publication.

## Conflicts of interest

None disclosed.

## References

[1] Swetter S.M., Johnson D., Albertini M.R. (2024). NCCN guidelines(R) insights: melanoma: cutaneous, version 2.2024. J Natl Compr Canc Netw.

[2] Bello D.M., Han G., Jackson L. (2016). The prognostic significance of sentinel lymph node status for patients with thick melanoma. Ann Surg Oncol.

[3] Swetter S.M., Thompson J.A., Albertini M.R. (2021). NCCN guidelines(R) insights: melanoma: cutaneous, version 2.2021. J Natl Compr Canc Netw.

[4] McMasters K.M., Chao C., Wong S.L. (2002). Interval sentinel lymph nodes in melanoma. Arch Surg.

[5] Egger M.E., Stevenson M., Bhutiani N. (2019). Should sentinel lymph node biopsy be performed for all T1b melanomas in the new 8(th) edition American Joint Committee on Cancer Staging System?. J Am Coll Surg.

[6] Miller J.R., Lo S.N., Nosrati M. (2023). Improving selection for sentinel lymph node biopsy among patients with melanoma. JAMA Netw Open.

[7] Munsch C., Lauwers-Cances V., Lamant L. (2014). Breslow thickness, clark index and ulceration are associated with sentinel lymph node metastasis in melanoma patients: a cohort analysis of 612 patients. Dermatology.

[8] Ma B., Gandhi M., Czyz S. (2025). Risk prediction models for sentinel node positivity in melanoma: a systematic review and meta-analysis. JAMA Dermatol.

[9] Greener J.G., Kandathil S.M., Moffat L., Jones D.T. (2022). A guide to machine learning for biologists. Nat Rev Mol Cell Biol.

[10] Li X., Zhao Y., Zhang D. (2023). Development of an interpretable machine learning model associated with heavy metals' exposure to identify coronary heart disease among US adults via SHAP: findings of the US NHANES from 2003 to 2018. Chemosphere.

[11] Collins G.S., Reitsma J.B., Altman D.G., Moons K.G. (2015). Transparent reporting of a multivariable prediction model for individual prognosis or diagnosis (TRIPOD): the TRIPOD statement. BMJ.

[12] Dhiman P., Ma J., Qi C. (2023). Sample size requirements are not being considered in studies developing prediction models for binary outcomes: a systematic review. BMC Med Res Methodol.

[13] Riley R.D., Ensor J., Snell K.I.E. (2020). Calculating the sample size required for developing a clinical prediction model. BMJ.

[14] van Buuren S., Groothuis-Oudshoorn K. (2011). Mice: multivariate imputation by chained equations in R. J Stat Softw.

[15] Lundberg S., Lee S.-I. (2017). A unified approach to interpreting model predictions. https://ui.adsabs.harvard.edu/abs/2017arXiv170507874L.

[16] Lo S.N., Ma J., Scolyer R.A. (2020). Improved risk prediction calculator for sentinel node positivity in patients with melanoma: the melanoma institute Australia nomogram. J Clin Oncol.

[17] Wong S.L., Kattan M.W., McMasters K.M., Coit D.G. (2005). A nomogram that predicts the presence of sentinel node metastasis in melanoma with better discrimination than the American Joint Committee on Cancer Staging System. Ann Surg Oncol.

[18] Fan X.J., Wan X.B., Huang Y. (2012). Epithelial-mesenchymal transition biomarkers and support vector machine guided model in preoperatively predicting regional lymph node metastasis for rectal cancer. Br J Cancer.

[19] Kriegeskorte N., Golan T. (2019). Neural network models and deep learning. Curr Biol.

[20] Juan C.K., Su Y.H., Wu C.Y. (2023). Deep convolutional neural network with fusion strategy for skin cancer recognition: model development and validation. Sci Rep.

[21] Tsiogka A., Rubin A.I., Gregoriou S., Soulaidopoulos S., Belyayeva H., Rigopoulos D. (2024). Prevalence of subungual melanoma in patients with cutaneous malignant melanoma: a systematic review and meta-analysis. J Eur Acad Dermatol Venereol.

[22] Valenzuela C.D., Fowler G., Kozuma K., Kusaka S., Vetto J.T. (2024). Long-term outcomes after amputation and sentinel node biopsy for subungual melanoma: a single-institution series. Am J Surg.

[23] Tapoi D.A., Gheorghisan-Galateanu A.A., Gosman L.M., Derewicz D., Costache M. (2024). The prognostic value of proliferative activity in cutaneous melanoma: a pilot study evaluating the mitotic rate and Ki67 index to predict patient outcomes. Biomedicines.

[24] Yerushalmi R., Woods R., Ravdin P.M., Hayes M.M., Gelmon K.A. (2010). Ki67 in breast cancer: prognostic and predictive potential. Lancet Oncol.

[25] Liang C., Li D., Liang Y. (2024). Prognostic and predictive significance of Ki67 in primary non-metastatic or recurrent acral melanoma: evidence from a multicenter retrospective study. Ann Surg Oncol.

[26] Tan S.X., Chong S., Rowe C. (2024). Lymphatic expression of the proliferation marker Ki67 is linked to sentinel node positivity, recurrence and mortality in primary cutaneous melanoma. Exp Dermatol.

[27] Gershenwald J.E., Scolyer R.A., Hess K.R. (2017). Melanoma staging: evidence-based changes in the American Joint Committee on Cancer eighth edition cancer staging manual. CA Cancer J Clin.

[28] Riquelme-Mc Loughlin C., Sandoval-Clavijo A., Blanco de Tord M. (2023). Prognostic role of microsatellites in melanoma and implications in the American Joint Committee on Cancer classification system: a cohort study. J Am Acad Dermatol.

[29] Kelly J.W., Sagebiel R.W., Calderon W., Murillo L., Dakin R.L., Blois M.S. (1984). The frequency of local recurrence and microsatellites as a guide to reexcision margins for cutaneous malignant melanoma. Ann Surg.

[30] Karakousis G.C., Gimotty P.A., Leong S.P. (2019). Microsatellitosis in patients with melanoma. Ann Surg Oncol.

[31] Stassen R.C., Maas C., van der Veldt A.A.M. (2024). Development and validation of a novel model to predict recurrence-free survival and melanoma-specific survival after sentinel lymph node biopsy in patients with melanoma: an international, retrospective, multicentre analysis. Lancet Oncol.

[32] Shim J.H., Shin H.T., Park J. (2017). Mutational profiling of acral melanomas in Korean populations. Exp Dermatol.

[33] Chang J.W. (2013). Acral melanoma: a unique disease in Asia. JAMA Dermatol.

[34] Gui J., Guo Z., Wu D. (2022). Clinical features, molecular pathology, and immune microenvironmental characteristics of acral melanoma. J Transl Med.

[35] Leacy F.P., Stuart E.A. (2014). On the joint use of propensity and prognostic scores in estimation of the average treatment effect on the treated: a simulation study. Stat Med.

[36] Riley R.D., Ensor J., Snell K.I. (2016). External validation of clinical prediction models using big datasets from e-health records or IPD meta-analysis: opportunities and challenges. BMJ.

[37] Reps J.M., Williams R.D., You S.C. (2020). Feasibility and evaluation of a large-scale external validation approach for patient-level prediction in an international data network: validation of models predicting stroke in female patients newly diagnosed with atrial fibrillation. BMC Med Res Methodol.

